# Low-frequency oscillations in coupled phase oscillators with inertia

**DOI:** 10.1038/s41598-019-53953-1

**Published:** 2019-11-22

**Authors:** Huihui Song, Xuewei Zhang, Jinjie Wu, Yanbin Qu

**Affiliations:** 1 School of New energy, Harbin Institute of Technology-Weihai, Weihai, Shandong 264209 China; 2 College of Engineering, Texas A&M University-Kingsville, Kingsville, Texas 78363 USA

**Keywords:** Energy grids and networks, Complex networks

## Abstract

This work considers a second-order Kuramoto oscillator network periodically driven at one node to model low-frequency forced oscillations in power grids. The phase fluctuation magnitude at each node and the disturbance propagation in the network are numerically analyzed. The coupling strengths in this work are sufficiently large to ensure the stability of equilibria in the unforced system. It is found that the phase fluctuation is primarily determined by the network structural properties and forcing parameters, not the parameters specific to individual nodes such as power and damping. A new “resonance” phenomenon is observed in which the phase fluctuation magnitudes peak at certain critical coupling strength in the forced system. In the cases of long chain and ring-shaped networks, the Kuramoto model yields an important but somehow counter-intuitive result that the fluctuation magnitude distribution does not necessarily follow a simple attenuating trend along the propagation path and the fluctuation at nodes far from the disturbance source could be stronger than that at the source. These findings are relevant to low-frequency forced oscillations in power grids and will help advance the understanding of their dynamics and mechanisms and improve the detection and mitigation techniques.

## Introduction

Coupled phase oscillators described by the Kuramoto model have been extensively studied to understand synchronization and other dynamic phenomena in complex systems^1,2^. Coupled phase oscillators with inertia, as an extension of the original Kuramoto model to the second order, have also received continuing research attention^3–22^. In early works^3,4^, mean field analysis showed that, in systems of globally coupled oscillators, the synchronization exhibits a first-order phase transition and there is hysteresis between two synchronized states. After including noise, similar phenomena were observed^5,6^, and the effects of noise on phase synchronization were revealed^7^. In the thermodynamic limit, analysis of the problem formulated as a Fokker-Planck-type equation of one-oscillator probability density^8^ uncovered rich phenomenology when oscillator’s natural frequency follows certain distributions^9^. More recent developments in this field include conditions for frequency synchronization under local coupling^10,11^, low-dimensional behavior in complex networks^12^, interplay between network topology and system dynamics^13–15^, different types of chimera states^16,17^, effects of other factors such as frustration^18^ and time delay^19^, nonlinear transient wave propagation prior to synchrony^20^, and bi-stability^21^ multi-stability^22^ patterns of synchrony.

Although significant progress has been made to understand conditions, transitions, and properties of synchronized states, the “reverse” processes, i.e., dynamics of desynchronization due to instability, noise, or external excitation, remain to be explored. The patterns and mechanisms of desynchronization have been discussed in the context of other networked systems^23–25^ and considered as an important feature of some neurophysiological processes^26,27^. While fluctuation-induced desynchronization in Kuramoto oscillators has been a recurrent research topic in the recent literature^28–32^, these studies mostly focus on the onset of desynchronization (i.e., stochastic escape). There are only a small number of works dedicated to the characterization of dynamical properties and evolution of desynchronized states under noise or external forcing. It is demonstrated in^33^ that desynchronization can be harnessed to reduce fluctuations. Interesting frequency response of a Kuramoto network driven by fluctuations has been analyzed in^34^. Statistical characteristics of the propagation of non-Gaussian fluctuations in the network have been obtained in^35^.

In this work, we analyze the response of second-order Kuramoto oscillator network motifs to sinusoidal forcing, the general model of which was first given in^5^. This problem is of specific relevance to power grid reliability and stability. On the one hand, modeling power grid as Kuramoto oscillator networks has driven a new interdisciplinary research thrust in the last decade^29,35–39^ (some studies were based on first-order Kuromoto model^40,41^). Following the initial model formulation^36^, a study demonstrated that decentralized power sources might be conducive to self-organized synchrony, which would enhance gird robustness^37^. New synchronization conditions were derived in terms of network topology and parameters to improve the practical applicability of the theoretical results to smart grid dynamics and control^38,39^. Despite the simplifying assumptions and limitations^37^, this approach roots in the conceptual framework based on the electromechanical model of rotating electric machines and thus able to generate illustrative and insightful results justifiable for real-world applications^42^.

On the other hand, in electric power engineering, the dynamics of the grid under substantial oscillatory disturbances (desynchronized states that may have catastrophic consequences such as blackouts) has been investigated from two perspectives. The first considers the propagation of disturbances in the grid in the form of low frequency (order of 0.1-1 Hz) electromechanical waves^43,44^. A continuum model was constructed to describe the traveling wave along transmission lines^43^, based on which a control method was proposed to damp the dynamics^45^. With the grid getting more and more extensive and complex, it is imperative to ensure that effective protection schemes are in place against damaging electromechanical waves^46^ and advanced techniques are used to monitor them^47^. The second perspective scrutinizes low-frequency (sometimes also called inter-area) oscillations observed at specific nodes^48–50^. Classical analysis based on equivalent oscillating circuit model^48^ explained how “natural” interaction among grid devices results in free oscillations. In contrast, forced oscillations, i.e., the grid (typically in the regime where free oscillations are inhibited) response to persistent external forcing, have become a growing concern in recent decades^50^, stimulating the development of detection algorithms^51^ and mitigation methods^52^.

The Kuramoto model is a promising alternative to the continuum model and the circuit model that not only captures disturbance propagation (like the continuum model) and forced oscillation (like the circuit model) in the grid but also sheds light on the effects of network topology (which continuum model is unable to show) and coupling strength (which circuit model does not clarify) on system dynamics. This work performs a numerical study of the desynchronization dynamics of some representative periodically-driven Kuramoto oscillator networks. In the context of this paper, desynchronization refers to a phase unlocked regime (the phase differences between nodes are no longer constant). As shown below, there is an “ac steady state” in which the phase of each node fluctuates at the same frequency as the external forcing around a state that deviates from the synchrony. Special attention is paid to the magnitude of this type of phase fluctuation and the effects of network and forcing parameters on the magnitude. In power grids, the knowledge of phase fluctuation magnitude at each node will be essential to the specification of its hosting capacity as well as necessary control measures.

## Model

The model in this work follows that of^36,37^, with the addition of sinusoidal forcing at a selected node. Consider a coupled network of *N* synchronous generators (production) and motors (load). Each node *i*(*i* = 1, …, *N*) can be described as a phase oscillator with its electromechanical phase ${\theta }_{i}=\Omega t+{\varphi }_{i}$, where Ω is the constant grid reference frequency and *ϕ*_*i*_ is the relative phase (or simply called phase here). For each oscillator, the generated (consumed) power should balance the power transmitted to (received from) the grid plus (minus) that for local acceleration and dissipation. Assuming that $|\frac{d{\varphi }_{i}}{dt}|\ll \Omega$, the governing equation of the system at node *i* is:

1 $$\frac{{d}^{2}{\varphi }_{i}}{d{t}^{2}}={P}_{i}-{\alpha }_{i}\frac{d{\varphi }_{i}}{dt}-\mathop{\sum }\limits_{j=1}^{N}\,{K}_{ij}\,\sin ({\varphi }_{i}-{\varphi }_{j})+A{\delta }_{il}\,[u(t-{t}_{1})-u(t-{t}_{2})]\,\sin \,(\omega t)$$

where *P*_*i*_ and *α*_*i*_ are the equivalent power and damping coefficient of the *i*-th oscillator, [*K*_*ij*_]_*N*×*N*_ is the matrix of coupling strength with each element *K*_*ij*_ being the product of the connectivity (1 if connected; 0 otherwise) and the coupling strength between nodes *i* and *j*, *A* and *ω* = 2*πf* are the magnitude and angular frequency of the external forcing applied at node *l* in time interval [*t*_1_, *t*_2_], *u*(*t*) is the unit step function, and *δ*_*il*_ is the Kronecker symbol. Equation (1) is solved using the 4^th^ order Runge-Kutta method.

In the un-disturbed case (*A* = 0), to reach synchrony, it is necessary that $\mathop{\sum }\limits_{i=1}^{N}\,{P}_{i}=0$ (energy balance). Further, in this work, the coupling strengths are so chosen that $\mathop{{\rm{\min }}}\limits_{j\leftrightarrow i}{K}_{ij}\ge |{P}_{i}|$ (*j*↔*i* means the two nodes are connected). This generally guarantees the existence of attractive fixed points (stable synchrony) of the system. It is also true in actual power grid since the capacity of transmission lines are by design higher than local generation or load. On the other hand, for each set of parameters (*P*_*i*_, *K*_*ij*_) used in the simulations, we numerically solve the un-disturbed steady state equation of Eq. (1) and confirm that the system has only one attractive fixed point. The regime with multiple fixed points is avoided in this study to make the simulation results comparable and without ambiguity.

Under these conditions, for each numerical case, the un-disturbed oscillators’ initial phases are chosen randomly from [0, 2*π*). Once all *ϕ*_*i*_’s reach equilibria, we record their equilibrium values as the initial conditions for solving Eq. (1). It is expected that before the application of external forcing, the system will remain in the initially synchronized state, and after the removal of forcing, the system will return to the initial state. Under the periodic forcing, the peak-to-peak magnitudes of all *ϕ*_*i*_’s fluctuations are measured to see how seriously an oscillator is affected. To describe the disturbance propagation, we record the starting time of each oscillator’s forced oscillation practically defined as the time when *ϕ*_*i*_ first crosses a pre-set value between the initial synchronized state and the time average of the ac steady state. By comparing the starting times of the oscillators, one can analyze the speed and path of propagation.

## Results

### Two-node networks

**Figure 1:**
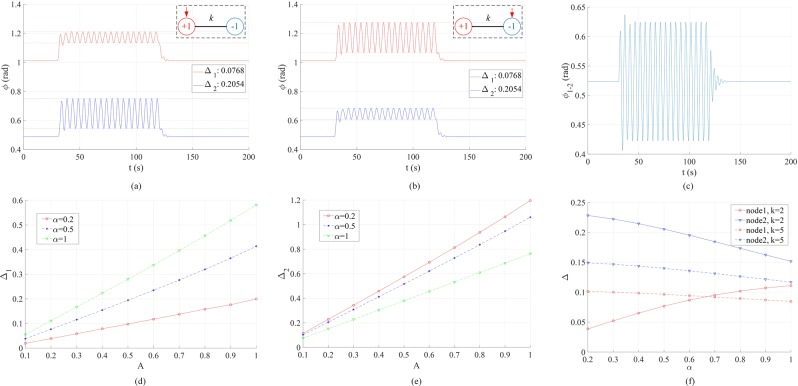
Phase fluctuation in a two-node network consisting of a generator (*P*_1_ =+ 1) and a load (*P*_2_ = −1) driven by an external periodic forcing *A* sin (2*πft*) where *f* = 0.2Hz. The forcing is added at *t* = 30 s and removed at *t* = 120 s. (**a**) Results when the forcing is applied to the generator node 1. Here *k* = 2, *α* = 0.5, *A* = 0.2. (**b**) Results when the forcing is applied to the load node 2 under the same conditions of (**a**). (**c**) The phase difference between the two nodes under the same conditions of (**a**). (**d**) Dependence of generator node phase fluctuation peak-to-peak magnitude Δ_1_ on the damping coefficient *α* and forcing amplitude *A* with *k* = 2. (**e**) Dependence of load node phase fluctuation peak-to-peak magnitude Δ_2_ on the damping coefficient *α* and forcing amplitude *A* with *k* = 2. (**f**) Dependence of phase fluctuation peak-to-peak magnitudes Δ_1,2_ on the damping coefficient *α* with *k* = 2 and 5 and *A* = 0.2.

We start with the two-node network due to its simplicity. When *P*_1_ = −*P*_2_ = *P*_0_, *α*_1_ = *α*_2_ = *α*, and *K*_12_ = *K*_21_ = *k*, Eq. (1) is reduced to $\frac{{d}^{2}\Delta \varphi }{d{t}^{2}}=2{P}_{0}-\alpha \frac{d\Delta \varphi }{dt}-2k\,\sin \,\Delta \varphi \pm A[u(t-{t}_{1})-u(t-{t}_{2})]\,\sin (\omega t)$ where $\Delta \varphi ={\varphi }_{1}-{\varphi }_{2}={\varphi }_{1-2}$, and the +/− means the forcing is applied at node 1/2. This is the equation of a damped nonlinear pendulum driven by an external force that has both dc (2*P*_0_) and ac (*A* sin (*ωt*)) components. Without the ac component, it can be shown that there exists an attractive fixed point at ${\varphi }_{1-2}=\arcsin ({P}_{0}/k)$ when $\frac{{P}_{0}}{k} < 1$. This is the regime under study in this work, i.e., there is a steady state with constant *ϕ*_1−2_. Usually this means both *ϕ*_1_ and *ϕ*_2_ are constants. Although, as shown in^37^, for smaller *k*’s and certain initial conditions, *ϕ*_1_ and *ϕ*_2_ may show identical oscillations which will be canceled in *ϕ*_1−2_, we do not consider this rare case and choose a higher *k* or an initial condition that leads to constant *ϕ*_1_ and *ϕ*_2_. Now with the ac component included, *ϕ*_1−2_ will fluctuate around its steady-state value. We numerically solve this problem, and some representative results are shown in Fig. 1.

Comparing Fig. 1(a,b), one can see that due to symmetry, the two cases (the forcing at node 1 and node 2) have the same pattern of phase fluctuation. The phase fluctuation has the same frequency as the external forcing. Figure 1(c) shows that *ϕ*_1−2_ also reaches an ac steady state with the same fluctuation frequency. This is an interesting result of relevance to the inter-area low-frequency oscillation in the power grid. The two nodes in Fig. 1 represent two grid zones where *ϕ*_1−2_ indicates the direction and amount of power flowing from node 1 to node 2. Under the conditions of Fig. 1, $\frac{{P}_{0}}{k} < 1$, which practically guarantees that *ϕ*_1−2_ does not have sustained free oscillations; however, with the periodic forcing, *ϕ*_1−2_ fluctuates around its normal operating point, which generally undermines the reliability and stability of a power grid. Note that the natural frequency of motion is $\sqrt{2k}$ (no damping or forcing). The resonant frequency of the damped driven system is lower than this value. The closer the external forcing frequency *f* is to the resonant frequency, the higher the magnitude of the fluctuation. In the case of Fig. 1, $\sqrt{2k}=2$ Hz; so the most interesting results are to be observed in the low frequency range, i.e., *f* ≲ 2 Hz. Higher frequency fluctuations are less important in terms of magnitude (see Fig. S1).

Further, in Fig. 1(d,e), we present the phase fluctuation peak-to-peak magnitudes (Δ_1,2_) at the two nodes with different damping coefficients (*α*) and forcing amplitudes (*A*). Keeping other conditions the same as Fig. 1(a), Δ_1,2_ increase about linearly with *A*, while increasing *α* results in higher Δ_1_ and lower Δ_2_. This counter-intuitive effect of damping is also plotted in Fig. 1(f), where, for comparison, the case of *k* = 5 is shown. Since this phenomenon can only be observed when *f* ≤ 0.2 Hz, its implication is that damping might not be effective for the reduction of very low-frequency phase fluctuations with relatively weak links. The detailed analysis will be reserved for continuing studies.

**Figure 2:**
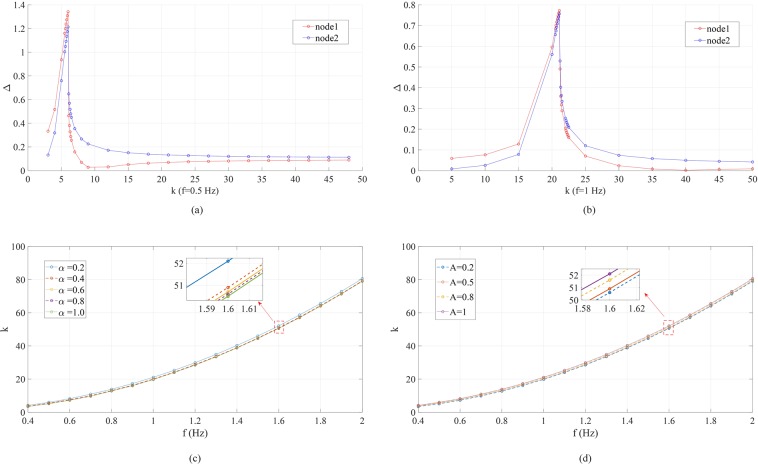
Coupling strength (*k*) dependence of the phase fluctuation in the two-node network studied in Fig. 1a. (**a**) Peak-to-peak fluctuation magnitudes Δ_1,2_ under different coupling strength *k* when *f* = 0.5Hz, *α* = 0.1, *A* = 0.2. (**b**) Peak-to-peak fluctuation magnitudes Δ_1,2_ under different **c**oupling strength *k* when *f* = 1Hz, *α* = 0.1, *A* = 0.2. (**c**) Dependence of the “optimal” coupling strength on the driving forcing frequency *f* with *A* = 0.2 and various values of *α*. (**d**) Dependence of the “optimal” coupling strength on the driving forcing frequency *f* with *α* = 0.1 and various values of *A*.

On the other hand, when *f* > 0.2 Hz, it is found that not only the dependency of Δ_1,2_ on *α* is similar to the case of *k* = 5 in Fig. 1(f), but also there is a “optimal” coupling strength *k* at which Δ_1,2_ peak. As shown in Fig. 2(a), when *f* = 0.5 Hz, both Δ_1_ and Δ_2_ reaches maxima at *k* ≈ 6. For smaller *k*’s, Δ_1_ > Δ_2_; for larger *k*’s, Δ_1_ < Δ_2_. Similar results are in Fig. 2(b) for *f* = 1 Hz, with the “optimal” coupling strength *k* ≈ 21. Note that in both cases, the forcing amplitude *A* = 0.2, and the peak Δ_1,2_ can be several times higher than *A*. Unlike the majority of literature on synchronization that requires some critical (minimum) coupling strength^38^, it is a new finding that, in a system as simple as the two-node network, there is another critical coupling strength under which the forced phase fluctuations resemble resonance. The difference is that here the variable is the coupling strength instead of the driving forcing frequency. In Fig. 2(c,d), we show that the “optimal” coupling strength *k* as a function of the forcing frequency *f* is insensitive to other factors such as *α* and *A*, implying that this phenomenon is due to the nonlinear interaction between the two nodes. Interestingly, Fig. 2 bears some similarity to the frequency response of forced Duffing oscillator^53^ (more details in *SI*, Note 1).

### Three- and four-node networks

**Figure 3:**
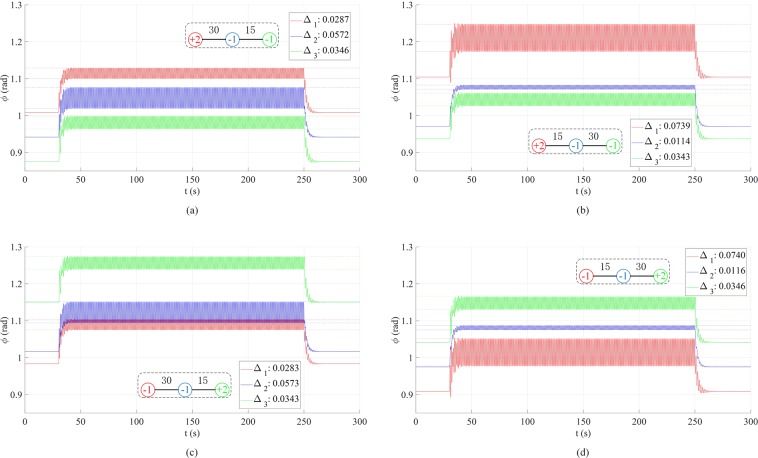
Phase fluctuation in the linear three-node network with driving forcing frequency *f* = 1Hz. The forcing is added to the left (red) node 1 at *t* = 30 s and removed at *t* = 250 s. The damping coefficient *α* = 0.5 and forcing amplitude *A* = 1. (**a**) Results when *P*_1_ =+ 2,*P*_2_ = *P*_3_ = −1,*k*_12_ = 30,*k*_23_ = 15. (**b**) Results when *P*_1_ =+ 2,*P*_2_ = *P*_3_ = −1,*k*_12_ = 15,*k*_23_ = 30. (**c**) Results when *P*_3_ =+ 2,*P*_2_ = *P*_1_ = −1,*k*_12_ = 30,*k*_23_ = 15. (**d**) Results when *P*_3_ =+ 2,*P*_1_ = *P*_2_ = −1,*k*_12_ = 15,*k*_23_ = 30.

We now move on to three-node (and four-node) systems. Figure 3 presents the phase fluctuation in a linear three-node network with forcing (*f* = 1 Hz) applied to node 1. Comparing the four cases, one concludes that 1) the nodal power distribution has an insignificant (<2%) effect on Δ’s ((a) versus (c), or (b) versus (d)), and 2) the variation in coupling strengths can lead to distinct patterns of Δ ((a) versus (b), or (c) versus (d)). To confirm the first point, we perform more simulations for confirmation (see Fig. S2). The second point is consistent with what we have seen in the two-node network. It is worth mentioning that these conclusions also hold for circular three-node network and four-node network with a branch (see Figs. S3 and S4). Physically, the nodal power serves as a dc driving force, so it is understandable that it has limited effect on the oscillatory motion. It is the interaction between nodes (characterized by the coupling strength) that “spread” the external forcing effects over the network.

**Figure 4:**
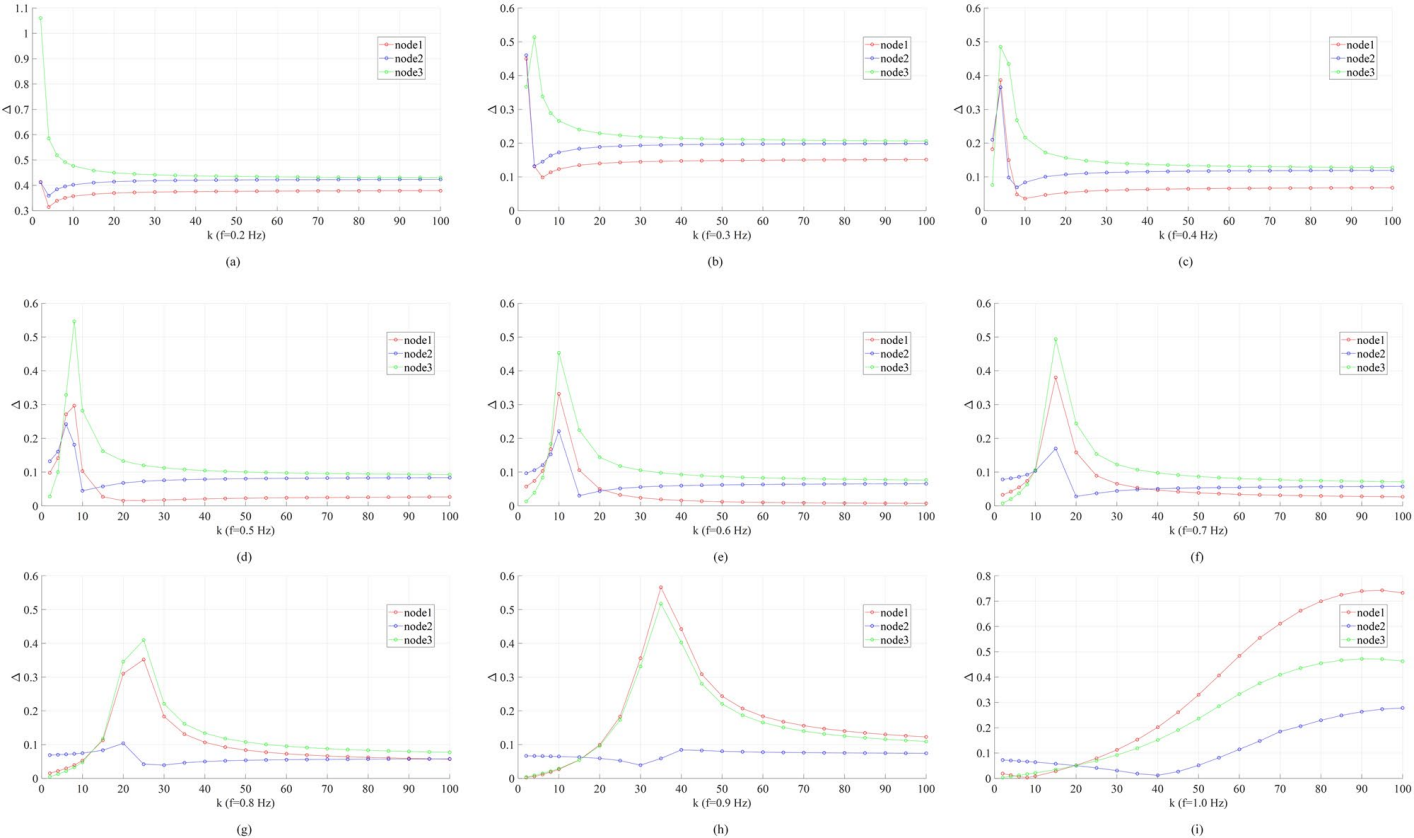
Coupling strength (*k*_23_) dependence of the phase fluctuation peak-to-peak magnitudes Δ_1,2,3_ in the linear three-node network studied in Fig. 3a under various driving forcing frequencies: (**a**) *f* = 0.2 Hz; (**b**) *f* = 0.3 Hz; (**c**) *f* = 0.4 Hz; (**d**) *f* = 0.5 Hz; (**e**) *f* = 0.6 Hz; (**f**) *f* = 0.7 Hz; (**g**) *f* = 0.8 Hz; (**h**) *f* = 0.9 Hz; (**i**) *f* = 1.0 Hz. Other parameters are: *k*_12_ = 30, *α* = 0.2, *A* = 1.

The “resonance” phenomena are also found in the linear three-node network. In Fig. 4, keeping *k*_12_ = 30 and varying *k*_23_, we plot the phase fluctuation peak-to-peak magnitudes Δ_1,2,3_ under different forcing frequencies. Similar to the two-node case, when *f* > 0.2 Hz, the “optimal” coupling strength starts to appear at which at least one node’s Δ peaks. The “optimal” coupling strength also increases with increasing *f*. For 0.4 ≤ *f* ≤ 0.7 Hz, we have Δ_3_ > Δ_1_ > Δ_2_ at the “optimal” coupling strength; increasing *f* to 0.9 Hz, Δ_1_ becomes the highest and Δ_2_ does not show an obvious peak near the “resonance” point. Note that the forcing is added at node 1. In power grids, to detect forced oscillations, it would be desired to monitor a node with maximum Δ. From Fig. 4, one can see that this node is one of the end nodes.

### Longer chains and rings

**Figure 5:**
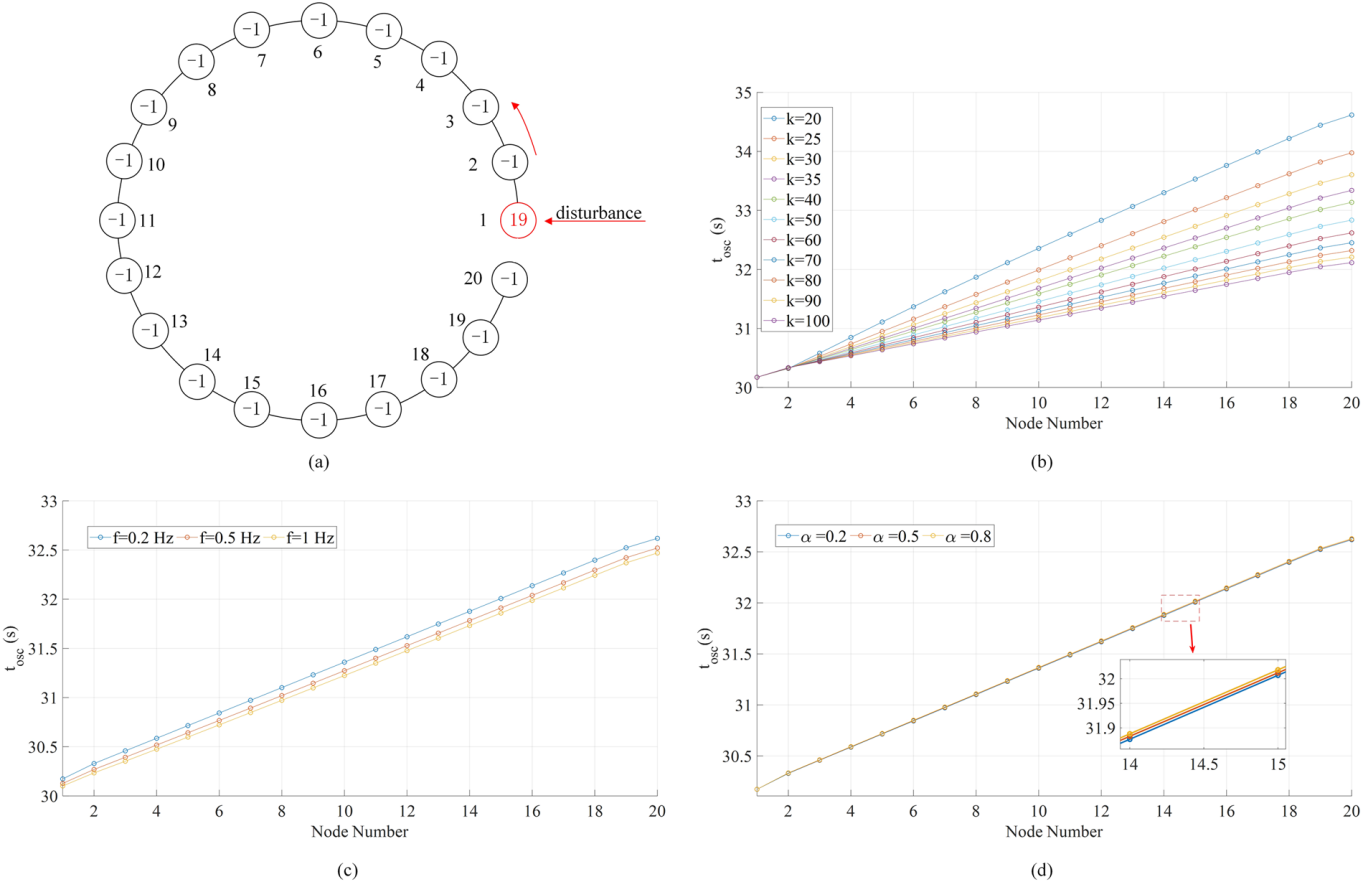
Phase fluctuation in a linear 20-node network driven by an external periodic forcing *A* sin (2*πft*). The damping coefficient *α* = 0.2 and forcing amplitude *A* = 1. (**a**) The network topology. The numbers in the circles indicate the power at the respective nodes. The forcing is added to the generator (red) node 1 at *t* = 30 s and removed at *t* = 250 s. (**b**) Propagation of phase oscillations along the chain with *f* = 0.2Hz and various global coupling strength *k*. The propagation is represented by the time the oscillation starts at each node. (**c**) Propagation of phase oscillations along the chain with *k* = 60an**d** different driving forcing frequencies *f*. (**d**) Propagation of phase oscillations along the chain with *k* = 60, *f* = 0.2Hz, and different damping coefficients *α*.

To study the forced oscillation in more complex systems, we consider the propagation of phase fluctuation as well as the distribution of phase fluctuation magnitudes. Figure 5(a) illustrates the configuration of a linear 20-node network with global coupling strength driven by external periodic forcing at node 1. Figure 5(b) shows the propagation of the phase oscillations along the chain. The higher the coupling strength *k*, the faster the phase oscillation propagates to the last node in the chain. After *k* is over 100, this trend comes to saturation. Due to the limitation of the Kuramoto model, this cannot be directly related to the speed of propagation since there is no length for each link. Nevertheless, one can still see that stronger links facilitates the propagation of low-frequency phase oscillations. Additional results in Fig. 5(c,d) show that the forcing frequency and the damping coefficient have negligible impacts on the propagation. In Fig. 5(c), the apparent delay in the *f* = 0.2 Hz case results from the method used to measure *t*_*osc*_.

**Figure 6:**
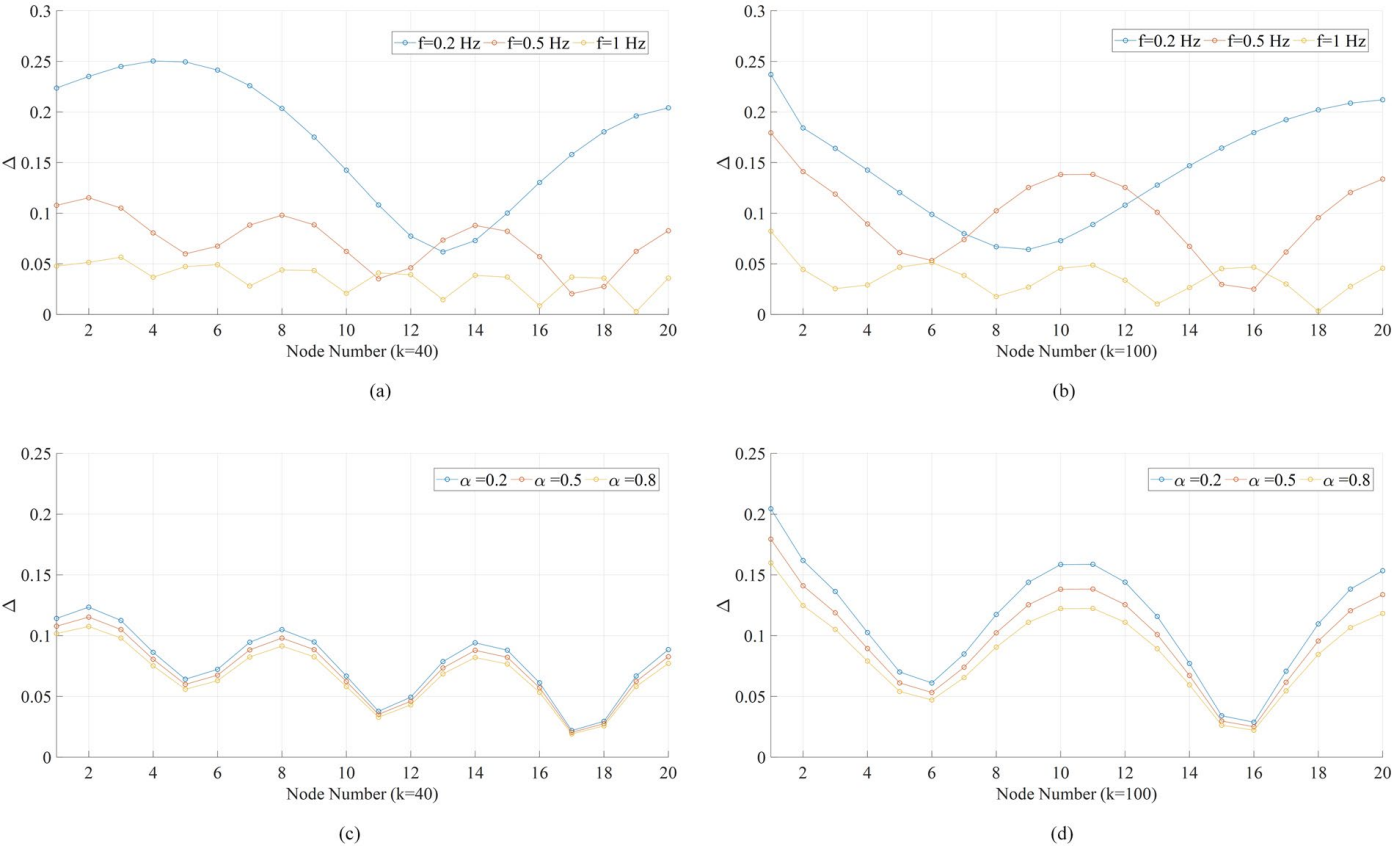
Phase fluctuation peak-to-peak magnitude (Δ) distributions in the linear 20-node network in Fig. 5. (**a**) Results when global coupling strength *k* = 40 and driving forcing frequency *f* = 0.2,0.5,1 Hz. The damping coefficient *α* = 0.5 and forcing amplitude *A* = 1. (**b**) Results when global coupling strength *k* = 100 and other parameters are the same as (**a**). (**c**) Results when global coupling strength *k* = 40, forcing amplitude *A* = 1, frequency *f* = 0.5 Hz, and damping coefficient *α* = 0.2,0.5,0.8. (**d**) Results when global coupling strength *k* = 100, and other parameters are the same as (**c**).

Figure 6 presents the distributions of Δ along the 20-node chain under various conditions. If Fig. 5 views the phase fluctuation as “traveling wave”, Fig. 6 displays something like “standing wave”. In Fig. 6(a), we find that the higher the forcing frequency, the more “bumps” there will be in the distribution of Δ. There is a decaying trend from node 1 to node 20, but the decay is not monotonic. Also in general, higher frequency corresponds to lower Δ. Comparing Fig. 6(b) with 6(a), one sees that the higher the coupling strength, the fewer “bumps” there will be in the distribution of Δ. Figure 6(c,d) indicate that the effect of the damping coefficient on the distribution of Δ is more significant under higher coupling strengths. Similarly, we obtain the distributions of ∆ along an *N*-node chain where 3 ≤ *N* ≤ 20 (Fig. S5). An interesting observation is that the fluctuation magnitude at node *N* is always a local maximum and in the cases of *N* ≥ 7, the value of ∆ at the 7th node counting backwards starting from node *N* is always a local minimum. In the cases of *N* ≥ 14, the value of ∆ at the 8th node counting backwards starting from this local minimum becomes a local maximum again. This pattern continues with longer chains and under different parameter settings (e.g., when *f* = 0.5 Hz, the numbers will be 3 and 4 instead of 7 and 8). The distribution of fluctuation magnitudes can be qualitatively inferred from the node that is the farthest from the forcing node, while the common sense may suggest the other direction. In Fig. S6, we plot the values of ∆ at node *N* in all the cases above, which display an oscillatory damping trend as the chain elongates.

**Figure 7:**
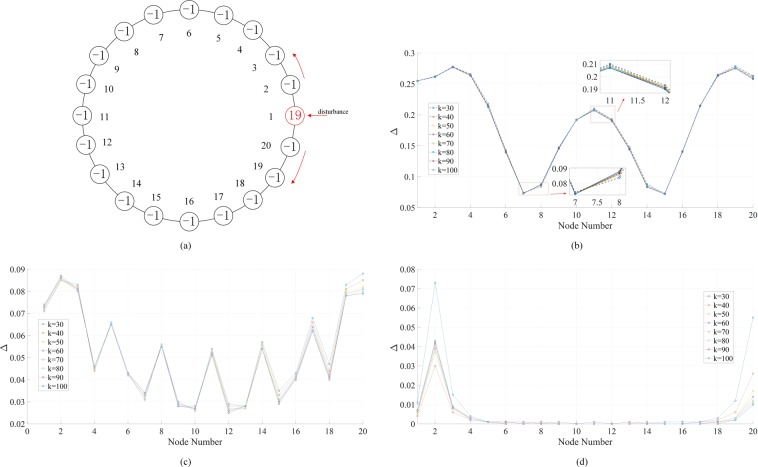
Phase fluctuation peak-to-peak magnitude (Δ) distributions in a circular 20-node network driven by an external periodic forcing *A* sin (2*πft*). The damping coefficient *α* = 0.2 and forcing amplitude *A* = 1. (**a**) The network topology. The numbers in the circles indicate the power at the respective nodes. The forcing is added to the generator (red) node 1. The coupling strength *k*_12_ = 40; all other coupling strengths except *k*_1,20_ are fixed at 10. *k*_1,20_ varies from 30 to 100. (**b**) Results when *f* = 0.2 Hz. (**c**) Results when *f* = 0.5 Hz. (**d**) Results when *f* = 1 Hz.

When a link is added between node 1 and node 20 in Fig. 5(a), the above network becomes a ring, as illustrated in Fig. 7(a). The resulting distributions of Δ are in Fig. 7(b–d). As expected, since there are now two pathways of propagation, the distributions of Δ show a decaying trend along both pathways from node 1 to node 11. The three sets of results point out that the higher the forcing frequency, the more difficult it can “penetrate” the network (most links with coupling strength *k* = 10). In the case of *f* = 1 Hz, the phase fluctuations are localized near nodes 1, 2, and 20. This phenomenon could inspire the design of power grids to damp the forced oscillations and improve system stability.

**Figure 8:**
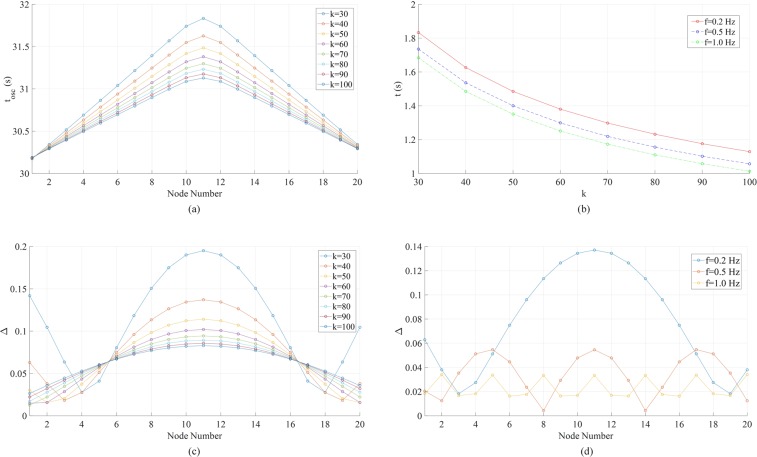
Phase fluctuation in the circular 20-node network in Fig. 7. The damping coefficient *α* = 0.2 and forcing amplitude *A* = 1. **(a)** Propagation of phase oscillations along the ring with *f* = 0.2 Hz and the global coupling strength *k* varying from 30 to 100. The propagation is represented by the time the oscillation starts at each node. **(b)** Effect of the global coupling strength *k* and forcing frequency *f* on the start time of the oscillation at the most distant node (node 11) after the application of the external forcing. **(c)** Phase fluctuation peak-to-peak magnitudes Δ with *f* = 0.2 Hz and various global coupling strength *k*. **(d)** Phase fluctuation peak-to-peak magnitudes Δ with global coupling strength *k* = 40 and different driving forcing frequencies *f*.

To make it comparable with Fig. 6, we simulate the forced oscillation in the ring now with global coupling strength *k*. Figure 8(a) shows the propagation along the ring. Figure 8(b) presents the propagation times from node 1 to node 11 (the farthest one from external forcing) under various *k*’s and *f*’s. Again, the forcing frequency does not affect how fast the phase fluctuations propagate. The higher the coupling strength, the faster the propagation. With *f* = 0.2 Hz and *k* in a range from 30 to 100, the distributions of Δ in the system are plotted in Fig. 8(c). Different from the chain in Fig. 6, the ring’s Δ distributions have similar profiles under various *k*’s (increasing *k* tends to flatten the profile), and node 11 always has the highest Δ. In addition, as shown in Fig. 8(d), the ring’s Δ distributions are symmetric along the two pathways. In Fig. S7, we show the results of a ring with 19 nodes and other conditions the same as Fig. 8(d). Now there are two farthest nodes (10 and 11) and the distribution features are very similar. Therefore the Δ distribution is not determined by even or odd number of nodes in the ring.

## Conclusions

This work considers a second-order Kuramoto model periodically driven at one node as the model of forced oscillations in power grids. Note that the un-forced system stays within the regime of stable synchrony and the external forcing does not introduce new dynamic regimes or cause regime changes. Motivated by the power grid application, we study the phase fluctuation magnitude at each node as well as the disturbance propagation in the network. There are three major findings. Firstly, given the network topology, coupling strength, the external forcing, and the node where the forcing is applied, the patterns of phase fluctuation change little with the node powers or damping coefficients; in other words, the phase fluctuation is primarily determined by the network structural properties and forcing parameters, not the parameters specific to individual nodes. Secondly, a new “resonance” phenomenon is discovered in which the phase fluctuation magnitudes peak when the coupling strength takes certain critical value. Note that the coupling strengths in this work are large enough to ensure the stability of equilibria in the unforced system. However, under the low-frequency forcing, the system with the critical coupling strength experiences much higher fluctuations, which has relevance and implications to the mechanisms of low-frequency forced oscillations in the power system. Finally, in the cases of long chain and ring-shaped networks, we show the disturbance propagation (its speed increased with coupling strength) and the distribution of the phase fluctuation magnitudes across the network. The Kuramoto approach captures an important but somehow counter-intuitive fact that the fluctuation magnitude distribution does not follow a simple attenuating trend and at some nodes far from the disturbance source, the fluctuation is even higher than that at the source.

This work has been an attempt to extend the previous studies on second-order Kuramoto oscillators from synchronization to one of the simplest desynchronized mode: ac steady state. It also contributes to the modeling and understanding of power grid forced oscillations based on the Kuramoto model. Further simulations can be conducted to investigate low-frequency oscillations under more realistic or complex network topologies. Theoretical analysis and development will be needed to provide a detailed and coherent explanation of the phenomena reported in this work.

## Supplementary information

Supplementary Information
