# Supplementary material for: Low-frequency oscillations in coupled phase oscillators with inertia
Source: Sci Rep. 2019 Nov 22;9:17414. doi: 10.1038/s41598-019-53953-1 (PMC6874549; doi:10.1038/s41598-019-53953-1)
Supplement: Supplementary file 1 — Supplementary Information [file 41598_2019_53953_MOESM1_ESM.docx]

**Low frequency oscillations in coupled phase oscillators with inertia**

**Supplementary Information**

Jinjie Wu^1,+^, Xuewei Zhang^2,+^, Yanbin Qu^1^, and Huihui Song^1,*^

^1^School of New energy, Harbin Institute of Technology-Weihai, Weihai, Shandong 264209, China

^2^ College of Engineering, Texas A&M University-Kingsville, Kingsville, Texas 78363, USA

**Figure S1**. Phase fluctuation peak-to-peak magnitudes corresponding to external forcing frequency $f\geq1$Hz in the two-node network of Fig. 1(a). The external forcing amplitude $A=1$.

**Figure S2**. Phase fluctuation in a linear three-node network driven by an external periodic forcing $A\sin(2\pi ft)$ with $f=0.1$Hz. The forcing is added to the left (red) node 1 at $t=30$ s and removed at $t=160$ s. Red, blue and green indicate node 1, 2 and 3, respectively. The numbers in the circles indicate the power at the respective nodes. The coupling strength $k=10$, damping coefficient $\alpha=0.5$ and forcing amplitude $A=1$. (**a**) Results when $P_{1}=+2, P_{2}=P_{3}=-1$. (**b**) Results when $P_{1}=-2, P_{2}=P_{3}=+1$. (**c**) Results when $P_{3}=-2, P_{2}=P_{1}=+1$. (**d**) Results when $P_{2}=-2, P_{1}=P_{3}=+1$.

**Figure S3**. Phase fluctuation peak-to-peak magnitudes $\Delta_{1,2,3}$ in the circular three-node network. Red, blue and green indicate node 1, 2 and 3, respectively. Here $f=0.5$ Hz, $\alpha=0.5$ , $A=1$. (a) Dependence on $k_{23}$ when $k_{12}=k_{13}=30$. (b) Dependence on $k_{23}$ when $k_{12}=10 , k_{13}=30$.

**Figure S4**. Phase fluctuation in a branching four-node network driven by an external periodic forcing $A\sin(2\pi ft)$ with $f=0.2$Hz. The forcing is added to the generator (red) node 1 at $t=30$ s and removed at $t=250$ s. The numbers in the circles indicate the power at the respective nodes. The damping coefficient $\alpha=0.2$ and forcing amplitude $A=1$. (**a**) Results when $P_{1}=+4, P_{2}=P_{4}=-1, P_{3}=-2, k_{12}=30, k_{23}=15, k_{24}=30$. (**b**) Results when $P_{1}=+4, P_{3}=P_{4}=-1, P_{2}=-2, k_{12}=30, k_{23}=15, k_{24}=30$. (**c**) Results when $P_{3}=-2, P_{2}=P_{1}=+1$. (**d**) Results when $P_{1}=+4, P_{2}=P_{4}=-1, P_{3}=-2, k_{12}=30, k_{23}=30, k_{24}=15$.

**Figure S5**. Phase fluctuation peak-to-peak magnitude ($\Delta$) distributions in linear $N$-node networks ($N$ from 3 to 20). The power at node 1 is $N-1$ and all other nodes have power $-1$. The sinusoidal forcing is applied at node 1. The damping coefficient $\alpha=0.2$, forcing amplitude $A=1$, frequency $f=0.2$ Hz, and global coupling strength $k=30$.

**Figure S6**. Phase fluctuation peak-to-peak magnitude ($\Delta$) at node $N$ in linear $N$-node networks ($N$ from 3 to 20) in Fig. S5. (a) The results when frequency $f=0.2$ Hz. (b) The results when frequency $f=0.5$ Hz.


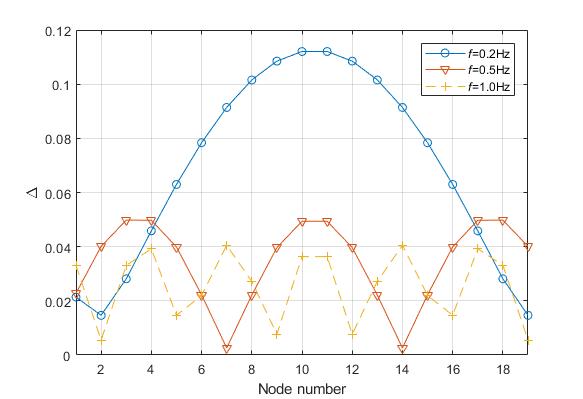


**Figure S7**. Phase fluctuation peak-to-peak magnitudes $\Delta$ in a 19-node circular network with $f=0.2, 0.5, 1$ Hz and global coupling strength $k=40$. The damping coefficient $\alpha=0.2$ and forcing amplitude $A=1$ (applied at node 1).

**Note S1**: Similarity between the forced two-node system and the forced Duffing oscillator

| Forced Duffing Oscillator | Forced Two-Node System |
| --- | --- |
| $\ddot{x}+\alpha\dot{x}+\beta x+\gamma x^{3}=A\sin(\omega t)$  Frequency response [53]: $z$ is the oscillation amplitude  $\left[ \left( \omega^{2}-\beta-\frac{3}{4}\gamma z^{2} \right)^{2}+{(\alpha\omega)}^{2} \right]z^{2}=A^{2}$ | $\ddot{\Delta\phi}+\alpha\dot{\Delta\phi}+2k\sin\Delta\phi=A\sin\left( \omega t \right)+2P_{0}$  Assuming small \|$\Delta\phi$\| and $\Delta\phi=x_{0}+x(t)$, then  $x_{0}\approx\arcsin(P_{0}/k)$ (dc part; see Fig. 1c)  $\ddot{x}+\alpha\dot{x}+2kx-\frac{k}{3}x^{3}+\cdots=A\sin\left( \omega t \right)$ (oscillatory part)  Frequency response:  $\left[ \left( \omega^{2}-2k+\frac{1}{4}kz^{2} \right)^{2}+{(\alpha\omega)}^{2} \right]z^{2}=A^{2}$  Since $z$ is small, approximately we have maximum $z$ when $\omega^{2}-2k=0$ or  $k=\frac{\omega^{2}}{2}=2\pi^{2}f^{2}$  For $f=1$ Hz, it yields $k=19.7$; for $f=0.5$ Hz, it yields $k=4.9$. The estimation is in good agreement with the results in Fig. 2.  In addition, we can see that the “optimal” coupling strength does not strongly depend on other parameters like $\alpha$ and $A$.  However, the above analysis has used very crude approximations. According to our simulations, both \|$\Delta\phi$\| and $z$ are not necessarily much smaller than 1. |
